# EBI-3 Chain of IL-35 Along With TGF-β Synergistically Regulate Anti-leishmanial Immunity

**DOI:** 10.3389/fimmu.2019.00616

**Published:** 2019-04-12

**Authors:** Mohammad Asad, Abdus Sabur, Mohammad Shadab, Sonali Das, Mohd. Kamran, Nicky Didwania, Nahid Ali

**Affiliations:** Infectious Diseases and Immunology Division, Council of Scientific and Industrial Research-Indian Institute of Chemical Biology (CSIR-IICB), Kolkata, India

**Keywords:** regulatory T cells, *Leishmania*, immune response, interleukin-35, transforming growth factor beta, immune suppression

## Abstract

Immunosuppression is a characteristic feature of chronic leishmaniasis. The dynamicity and the functional cross talks of host immune responses during *Leishmania* infection are still not clearly understood. Here we explored the functional aspects of accumulation of immune suppressive cellular and cytokine milieu during the progression of murine visceral leishmaniasis. In addition to IL-10 and TGF-β, investigation on the responses of different subunit chains of IL-12 family revealed a progressive elevation of EBI-3 and p35 chains of IL-35 with *Leishmania donovani* infection in BALB/c mice. The expansion of CD25 and FoxP3 positive T cells is associated with loss of IFN-γ and TNF-α response in advanced disease. *Ex-vivo* and *in vivo* neutralization of TGF-β and EBI-3 suggests a synergism in suppression of host anti-leishmanial immunity. The down-regulation of EBI-3 and TGF-β is crucial for re-activation of JAK-STAT pathway for induction as well as restoration of protective immunity against *L. donovani* infection.

## Introduction

Maintenance of immunological self-tolerance and homeostasis by restraining disproportionate and detrimental immune responses is primarily mediated by regulatory cytokine secreting lymphocytes (1). Conversely, expansion of regulatory cellular and cytokine milieu may lead to compromised immunity against certain infections such as *Brucella*, HIV, helminthes, and *Mycobacteruim tuberculosis* including antitumor host immune responses (2–5). However, the correlation between effector and regulatory cell populations especially in terms of sensing and secretion of cytokines during diseased condition is still not well understood (6).

Visceral leishmaniasis (VL) is a potentially lethal disease caused by parasitisation of cellular components of innate immune system by *Leishmania donovani/Leishmania infantum* (7). A dysfunctional cell mediated immune response is one of the characteristic features of chronic VL (8, 9). Several studies have suggested the role of IL-10 and TGF-β in subversion of proinflammatory response in active VL (10, 11). Despite crucial evidences of the role of these cytokines in augmenting VL pathology, the mode of action of these immunosuppressive cytokines is not clearly understood (12). Apart from IL-10 and TGF-β, the role of other immunosuppressive cytokines in VL is yet to be established. IL-35 has been reported for its immunosuppressive activity in autoimmunity and infectious diseases (13–15). IL-35 is a heterodimeric cytokine with two polypeptides “α” and “β” chains. These polypeptides may participate in the construction of two or more cytokines for example the α-chain, p35, is shared by IL-12 and IL-35, and the β-chain EBI-3 is shared by IL-27 and IL-35 (15). The roles of the subunit chains of IL-35 in immuno-regulation during VL are not known.

In the present study, we show the dynamicity of different subsets of CD4^+^ T cells at different stages of *L. donovani* infection of BALB/c mice and their contribution to different cytokine responses. In consistence to the previous reports of IL-10 and TGF-β, we additionally report the effect of the subunit chains of IL-35. We demonstrated the mechanistic action of TGF-β and EBI-3 in inhibition of CD4^+^ T cell-mediated Th1 response and its consequent effect on VL pathogenesis.

## Materials and Methods

### Animals and Parasites

BALB/c mice (6–8 weeks old), bred in the Institute's animal house facility, were infected with *L. donovani* strain AG83 (MHOM/IN/1983/AG83) (ATCC No. ATCC® PRA-413™), originally isolated from an Indian kala-azar patient, was maintained by serial passage in hamsters.

### Determination of Hepatic and Splenic Parasite Burden

Spleens from infected hamsters were aseptically removed and the splenocytes were cultured in Schneiders' insect medium containing 10% FBS and 1% penicillin streptomycin to allow the infected amastigotes to transform into promastigotes at 22°C for 2 weeks. The freshly transformed promastigotes were then cultured in M199 medium containing 10% FBS and 1% penicillin streptomycin and passaged in fresh medium maintaining a parasites density of 10^6^ promastigotes/ml. The promastigotes of 2nd or 3rd passage were washed several times in 0.02 M phosphate-buffered saline (PBS) of *p*H 7.2 and observed in the microscope before injecting (2.5 × 10^7^ cells/0.2 ml PBS) *i.v*., into BALB/c mice as described previously (16). At different mentioned time points of infection, spleen and liver were removed from the mice and multiple impression smears were prepared and stained with Giemsa. Organ parasite burdens expressed as Leishman-Donovan units (LDU) were calculated as the number of parasites per 1,000 nucleated cells × organ weight (mg). To determine whether the spleen and liver contained live parasites, the parasite burden was quantified in these tissues by Limiting Dilution Assay (LDA) as previously described (17–19). Briefly, a weighed small piece of spleen or liver from experimental mice was first homogenized in Schneider's medium supplemented with 10% FCS, and then diluted with the same medium to a final concentration of 1 mg/ml. Five-fold serial dilution of the homogenized tissue suspensions were plated in 96-well plates and incubated at 22°C for 2–3 weeks. Wells were examined for viable and motile promastigotes after 15 days, and the reciprocal of the highest dilution positive for parasites was considered as the parasite concentration per milligram of tissue. The total organ parasite burden was calculated using the weight of the respective organs.

### Cell Proliferation and Cytokine Assays

For cell proliferation and cytokine assays, single cell suspensions of infected and control mice splenocytes were prepared in RPMI 1640 supplemented with 10% FBS, l00 U/ml penicillin G sodium, 100 μg/ml streptomycin sulfate and 50 μM β-mercaptoethanol (complete medium). RBCs were removed by lysis with 0.14 M Tris-buffered NH_4_Cl. The remaining cells were washed twice with culture medium and viable mononuclear cells were counted in a haemocytometer by Trypan blue exclusion method. The cells were then cultured in a 96 well flat bottom plate at a density of 2 × 10^5^ cells/well in a final volume of 200 μl complete medium and stimulated with Leishmanial-antigen (LAg) (10 μg/ml). The cells were incubated for 72 h at 37°C in a humified chamber containing 5% CO_2_ as described earlier with little modifications (20). For cytokine analysis, culture supernatants were collected after 72 h of incubation and the concentration of IL-12p40, TNF-α, IFN-γ, IL-12p35, IL-4, IL-10, TGF-β, EBI-3 (USCN Life Science), and IL-27p28 (eBioscience) were quantified by ELISA in accordance with the manufacturer's instructions. All the kits were purchased from Beckton Dickinson (BD Pharmingen), unless mentioned. Sera were collected from various groups of mice, as required and cytokine ELISA was performed according to manufacturers' instructions.

### Flow Cytometry

Splenocytes from different groups of experimental and healthy mice were stimulated for 12 h with LAg (10 μg/ml) as described earlier (20). Brefeldin A (10 μg/ml) was added to the cultures 2 h before harvest. The cells were then washed in FACS buffer (0.02 M PBS, 1% FBS, and 0.01% sodium azide) and stained with fluorescent conjugated surface markers (0.2 μg/sample) for CD3 (APC-Cy7), CD4 (PE-Cy7 or BUV395), CD8 (Percp-Cy5.5), and CD25 (FITC). Subsequently, cells were permeabilized with FACS permeabilizing solution and washed in FACS buffer containing 0.1% saponin. The cells were then stained for intracellular marker, FoxP3 (Alexa Fluor 647) and cytokines, IL-4 (PECF594), IL-10 (BV711), TGF-β (eFluor710, eBioscience or BV421), EBI-3 (PE, BioLegends), TNF-α (BV650), and IFN-γ (Pacific blue). All the kits were purchased from BD Pharmingen, unless mentioned. Data were acquired by LSR Fortessa (BD Biosciences) and analyzed through FlowJo software (TreeStar). Fluorescence Minus One (FMO) for cytokine or rare cell populations was used for elimination of false positive signal in multiparametric study.

### Co-culture of CD4^+^CD25^−^ and CD4^+^CD25^+^ T Cells

Four months infected BALB/c mice were sacrificed and spleen were removed aseptically. CD4^+^CD25^−^ and CD4^+^CD25^+^ T cells were enriched by magnetic sorting according to manufacturer's protocol using lymphocyte enrichment kits (BD Pharmingen) (21). CD4^+^CD25^−^ T cells, labeled with carboxyfluorescein succinimidyl ester (CFSE) **(**Molecular Probes) were stimulated with anti-CD3 (10 μg/ml) and anti-CD28 (10 μg/ml) in 96-well plates and cultured with CD4^+^CD25^+^ T cells in various ratios (keeping total cells numbers 5 × 10^5^/ well) (13). After 96 h cells were collected for CFSE based CD4^+^CD25^−^ proliferation assay through flow cytometry. In few experiments, neutralizing mAbs (BioLegend) against CD25, IL-27(p28), IL-10, TGF-β and EBI-3 were added (20 μg/ml each) in the co-culture of CD4^+^CD25^−^ T cells and CD4^+^CD25^+^ T cells (1:1 ratio) and post 96 h, the culture supernatants were collected for cytokine ELISA.

### *In vivo* Neutralization of TGF-β and IL-35

TGF-β and IL-35 (EBI-3) were neutralized by mAbs (BioLegend) as reported (22, 23) with little modifications. Briefly, EBI-3 and TGF-β antibodies were administered in 200 μl PBS solution (0.02 M, *p*H 7.2) into the peritoneum of the BALB/c mice either individually (200 μg/mice/antibody) or in combination (100 μg/mice/antibody) 24 h prior to infection and subsequently each week doses until sacrificed post infection. Separate groups of isotype control (200 μg/mice IgG cocktail) and α-CD25 antibodies (200 μg/mice) were also included.

### Western Blot

Splenic cells were processed for cell lysate preparation, as described earlier (24). Firstly cells were lysed in cell lysis buffer (1X TBS containing 1% TritonX-100, 1 mM DTT, protease and phosphatase inhibitor cocktail), and the protein concentration in the cleared supernatants obtained after centrifuging at 1000 g were estimated using Lowry's method. The cell lysates were then resolved by 10–12% SDS-PAGE and transferred to nitrocellulose membrane (Millipore). The membranes were then blocked with 5% BSA in TBS for 1 h at room temperature and probed with primary Ab diluted in 5% BSA in TBS by incubating at room temperature for 2 h. After washing the membranes with TBST (Tween 0.5%) thrice for 5 min each, they were again probed with secondary antibody in TBST for 1 h. The membranes were then washed again three times each with TBST and finally detected by chemiluminescence.

### Statistical Analysis

All data comparisons were tested for significance by One-way analysis of variance (ANOVA) and Tukey's multiple comparisons using Graph Pad Prism version 5.0 (GraphPad Software, v. 5.0, (or Student's T test were employed were ever indicated) San Diego, CA). Results with *p* < 0.05 were considered to be statistically significant.

## Results

### Progression of *L. donovani* Infection and Associated Cytokine Responses in BALB/c Mice

**Figure 1:**
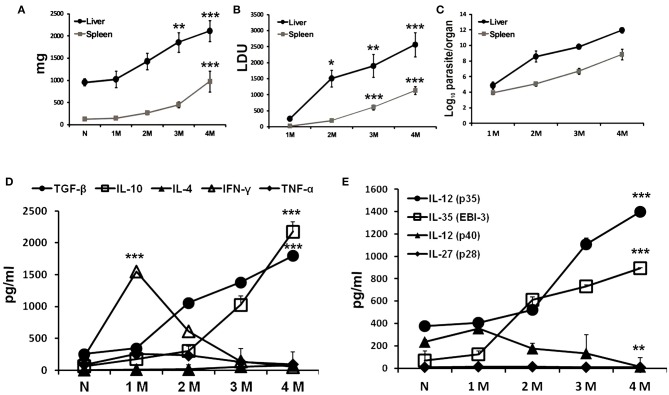
Progressive *L. donovani* infection synchronized with immuno-suppression in BALB/c mice. BALB/c mice, infected i.v. with 2.5 × 10^7^ *L. donovani* amastigotes, were sacrificed at indicated times post infection. Total organ weights of liver and spleen **(A)** as well as their parasite burden were determined by LDU **(B)** and LDA **(C)**. Cytokines profile of LAg (10 μg/ml) stimulated culture supernatants of splenocytes obtained from naïve and infected mice were obtained for IFN-γ, TNF-α, IL-4, IL-10, and TGF-β **(D)**, and the subunit chains of IL-12 family IL-12 (p35), IL-35 (EBI-3), IL-12 (p40), and IL-27 (p28) **(E)** by ELISA. Data expressed as means ± SE for six mice per group are representative of at least three independent experiments with similar results. *P < 0.05; **P < 0.01; ***P < 0.001 as assessed by one-way ANOVA and Tukey's multiple comparison tests.

Sets of BALB/c mice infected at different time points with *L. donovani* including uninfected controls were sacrificed to evaluate organomegaly, parasite burden and infection induced immunomodulations. Progressive increase in weight of liver and spleen was apparent during the course of infection (Figure 1A). No apparent organomegaly were observed in age matched control groups. Infection induced hepato-splenomegaly was directly correlated to the parasite burdens in the respective organs as estimated by LDU as well as LDA methods (Figures 1B,C). A non-resolving infective pattern was observed until 4 months of infection (liver LDU = 2558 ± 374.9; log_10_ values of LDA = 11.94 ± 0.36 and spleen LDU = 949.9 ± 123; log_10_ values of LDA = 8.84 ± 0.68). Cytokine responses in infected mice were assayed from culture supernatants of leishmanial-antigen stimulated splenocyte cultures. Like natural infection, a gradual surge in the levels of IL-4, IL-10, and TGF-β were observed, while the levels of IFN-γ and TNF-α increased gradually at early infection but were reduced to basal level at chronic infection (Figure 1D). Among various Chains of IL-12 family, the expressions of IL-12 (p35) and EBI-3 were progressively elevated with infection, whereas, the expression of IL-27 (p28) chain was found unaltered (Figure 1E). Besides, IL-12 the expression of IL-12 (p40) chain was down-regulated at chronic infection.

### Immunophenotyping of Cytokine Producing CD4^+^ T Cells in Infected Mice

**Figure 2:**
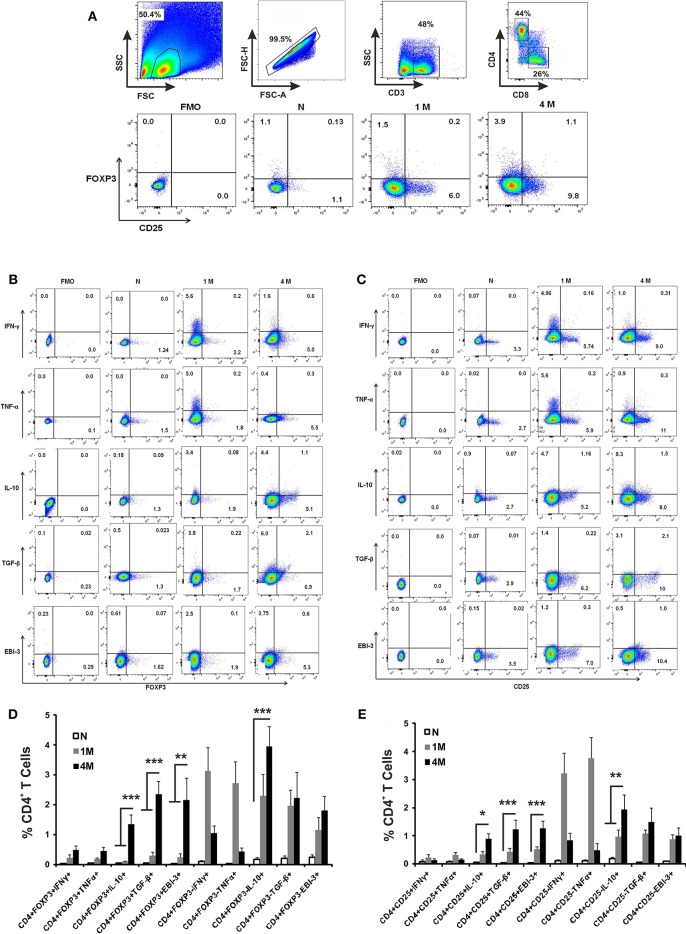
Role of CD4^+^ T cell subsets in cytokine production during *L. donovani* infection. Infected and control mice splenocytes were stimulated with Leishmanial membrane antigens (LAg) (10 μg/ml) overnight and then cells were processed for flow cytometric studies. CD3^+^ T cells were gated from single cell population of splenic lymphocytes, followed by CD4 and CD8 cells **(A)**. Subsequently, sub-sets of CD4 cells were gated based on Fluorescence Minus two controls for the identification of IFN-γ, TNF-α, IL-10, TGF-β and EBI-3 positive cells among CD4^+^Foxp3^+^ **(B)** as well as CD4^+^CD25^+^ **(C)** as representative dot plots. Data are expressed as means ± SE of percentage of cytokine producing CD4^+^Foxp3^+^/ CD4^+^Foxp3^−^ T cells **(D)**, and similarly of CD4^+^CD25^+^/ CD4^+^CD25^−^ T cells **(E)**, for six mice per group with three experimental repeats. Student's *T*-tests were applied to compare the differences in percentage proportion of cytokine producing cells as compared to uninfected controls. *P < 0.05; **P < 0.01; ***P < 0.001.

Splenocytes obtained from naïve and infected mice were immuno-phenotypically characterized for cytokine producing CD4^+^ T by flowcytometry. CD4^+^ T cells were gated from CD3^+^ single cell population of splenic lymphocytes (Figure 2A). CD4^+^ T cells were studied for the proportion of CD4^+^CD25^+^ and CD4^+^FoxP3^+^ and combination of both. The phenotypically characterized CD4^+^ T cell populations were analyzed for IFN-γ^+^, TNF-α^+^, IL-10^+^, TGF-β^+^, and EBI-3^+^ T cells. Fluorescence Minus One (FMO) controls were used to rule out false positive populations. The study reveals that the percentage of CD4^+^CD25^+^ and CD4^+^ FoxP3^+^ T cells were progressively enhanced until 4 months of infection compared to control naïve mice (Figure 2A). Analysis of cytokine secretion from early (1 month) infected CD4 T cells reveals an enhanced population of IFN-γ+, TNF-α^+^, IL-10^+^, TGF-β^+^, and EBI-3^+^ T cells compared to uninfected controls (Figures 2B,C). CD4^+^ T cells from late (4 months) infected mice show a diminished population of IFN-γ+, TNF-α^+^ as compared to early infected CD4^+^ T cell populations, while the IL-10^+^, TGF-β^+^, and EBI-3^+^ T cells show proportionate surge with disease progression (Figures 2B,C). The study further reveals that while production IFN-γ and TNF-α are dominantly restricted to CD4^+^FoxP3^−^ and CD4^+^CD25^−^ T cells, the sources of IL-10, TGF-β, and EBI-3 are from both positive and negative populations of FoxP3 and CD25 (Figures 2B–E).

### Increased Proportion of CD4^+^CD25^+^ T Cells *ex vivo* Leads to Loss of Cell Proliferation and Induction of Regulatory Cytokines

**Figure 3:**
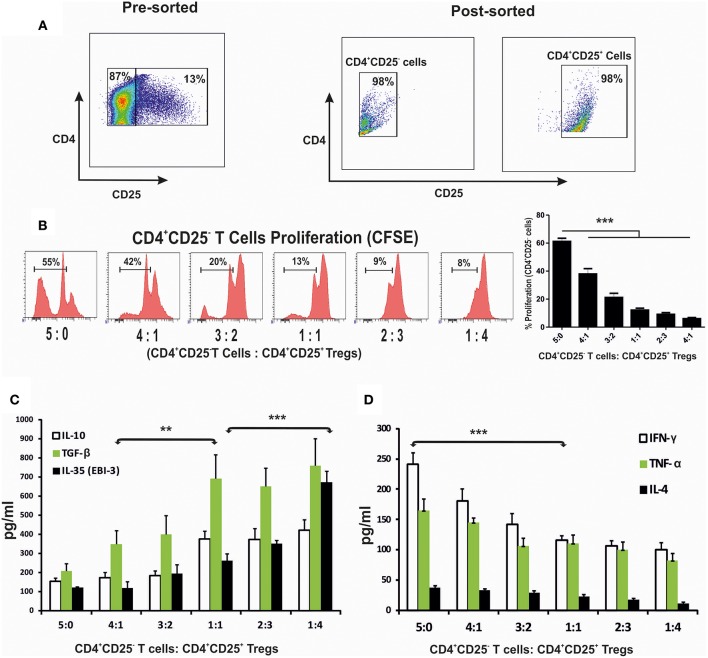
CD4^+^CD25^+^ T cells suppressed protective immune response when co-cultured with CD4^+^CD25^−^ T cells. Splenic CD4^+^CD25^−^ T cells (labeled with CFSE) and CD4^+^CD25^+^ T cells, were enriched through magnetic sorting from 4 months infected mice **(A)**, were stimulated with anti-CD3 and anti-CD28 and co-cultured in 96 U bottom plate at various indicated ratios. After 96 h cells were collected for CFSE based CD4^+^CD25^−^ proliferation assay through flow cytometry. Histograms and graph show percent proliferation of CD4^+^CD25^−^ cells co-cultured with CD4^+^CD25^+^ T at various ratios **(B)**. Supernatants of the co-culture were used for cytokine profiling **(C,D)**. Data expressed as means ± SE for at least five mice per group are representative of three independent experiments with similar results. **P < 0.01; ***P < 0.001 as assessed by one-way ANOVA and Tukey's multiple comparison tests.

To evaluate how CD4^+^ cells and their cytokine responses are associated with the disease progression, splenic CD4^+^CD25^−^ and CD4^+^CD25^+^ T cells of 4 months infected mice were enriched (Figure 3A). Co-cultures of CD4^+^CD25^−^ T cells with CD4^+^CD25^+^ T in various proportions in presence of the anti-CD3 and anti-CD28 stimulation were assayed for CFSE labeled cell proliferation as well as cytokine responses in culture supernatants. A significant loss in the cell proliferative responses were observed with increasing proportions of CD4^+^CD25^+^ T cells (Figure 3B). At equal concentration of CD4^+^CD25^−^ and CD4^+^CD25^+^ T cells (1:1 ratio), T cell proliferation was suppressed to ~12%. Study of the cytokine response in the co-culture supernatants showed that IL-10, TGF-β and EBI-3 increased while IFN-γ and TNF-α levels decreased with the increasing CD4^+^CD25^+^ T cells (Figures 3C,D). Moreover, IL-10, TGF-β, and EBI-3 secretion was found to be elevated with the increase in CD4^+^CD25^+^ T cells in co-culture. However, there was unnoticeable deflection in the levels of IL-4 at various ratios of CD4^+^CD25^−^ and CD4^+^CD25^+^ T cells.

### Neutralization of Regulatory Cytokines *ex vivo* Restores Cell Proliferation and Proinflammatory Cytokine Response

**Figure 4:**
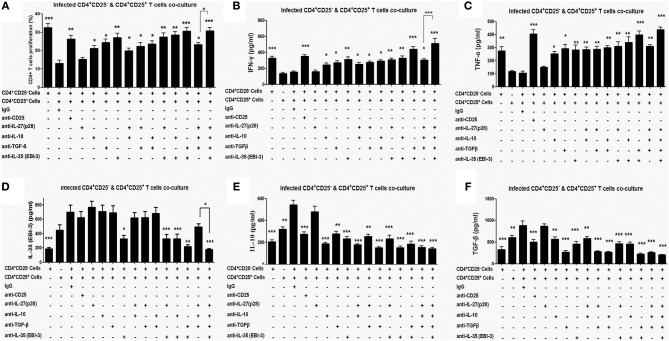
Proliferative and cytokine responses of CD4^+^CD25^−^ T cell, co-cultured with CD4^+^CD25^+^ T, in the presence of neutralizing antibodies. Indicated neutralizing antibodies were added (20 μg/ml each) in the co-culture of splenic CD4^+^CD25^−^ T cells (CFSE labeled) and CD4^+^CD25^+^ T cells (1:1 ratio) obtained from 4 months infected mice (as described in Materials and Methods). After 96 h cells were processed for CFSE based cell proliferation assay **(A)** and the culture supernatants were collected for estimation of cytokines, IFN-γ **(B)** TNF-α **(C)** IL-10 **(D)**, TGF-β **(E)** and EBI-3 **(F)**. Data represented as means ± SE for five mice per group are representative of three independent experiments with similar results. *P < 0.05; **P < 0.01; ***P < 0.001 between the neutralized and IgG control groups were assessed by two tailed Student's *T*-test.

The co-cultures of CD4^+^CD25^−^ and CD4^+^CD25^+^ T cells were treated with neutralizing monoclonal antibodies (mAbs) against CD25, IL-27 (p28), IL-10, TGF-β, and EBI-3 either singly or in indicated combinations. Neutralization with αCD25 antibodies in the co-culture of CD4^+^CD25^+^ and CD4^+^CD25^−^ T cells from infected mice leads to restoration of cellular proliferation (Figure 4A). Administration of αIL-10, αEBI-3 or αTGF-β resulted in partial gain in cell proliferation as compared to IgG controls. Moreover, there was no supplementary effect of adding various combinations of αIL-27 (p28), αIL-10, and αTGF-β. Interestingly, neutralization of EBI-3 either alone or in combination with TGF-β show proportionate restoration of cell proliferation in the stimulated co-cultures.

Similar to proliferative responses, neutralization with αIL-10, αEBI-3, or αTGF-β leads to partial but significant restoration of IFN-γ and TNF-α level in the stimulated co-culture (Figures 4B,C). Neutralization of IL-27 (p28) was apparently ineffective. Among the combined neutralization of various cytokines, administration of αEBI-3 along with αTGF-β resulted in the most effective boost of IFN-γ and TNF-α response (*p* < 0.001), followed by treatment of αEBI-3 along with αIL-10 (*p* < 0.01).

Effects of neutralization of CD25, IL-10, TGF-β, EBI-3, and IL-27 (p28) on disease promoting cytokine response were also examined. While homologous suppression following neutralization was observed for all the cytokines, cross regulation of cytokine response could be observed for TGF-β and EBI-3 neutralization on IL-10 production (Figures 4D–F). Production of TGF-β was regulated by neutralization of EBI-3 and vice versa (Figures 4D–F).

### Administration of αEBI-3 Along With αTGF-β in the Infected Mice Boosted Host Protective Response Concurrent With a Decline in the Disease Promoting Cytokines

**Figure 5:**
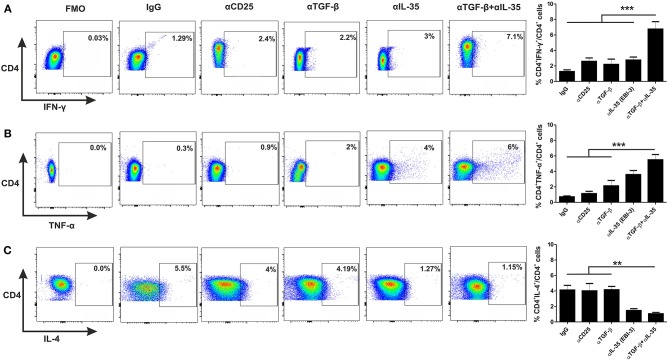
Administration of neutralizing antibodies promotes proinflammatory cytokine producing CD4^+^ T cells. Infected mice treated with various neutralizing mAbs were sacrificed and their splenocytes were stimulated with LAg (10 μg/ml) and cultured. After 24 h, cells were processed for FACS studies as described in Materials and Methods. Percent population CD4^+^ T cells producing IFN-γ **(A)**, TNF-α **(B)**, and IL-4 **(C)** were studied post acquisition of the data. Data expressed as means ± SE for five mice per group are representative of two independent experiments with similar results. **P < 0.01; ***P < 0.001 as assessed by one-way ANOVA and Tukey's multiple comparison tests.

For investigation of the involvement of EBI-3 and TGF-β with the disease progression, monoclonal αEBI-3 and αTGF-β antibodies were administered into the peritoneal cavity of the mice either individually or in combination. The first dose was administered 24 h prior *Leishmania* infection. Subsequent doses were given weekly until the mice were sacrificed. One group of infected mice was also administered with the isotype control (IgG) and another group was treated with αCD25 antibodies. After 3 months of infection, mice were sacrificed and LAg stimulated splenocytes were cultured for immunophenotyping and cytokine response. IFN-γ and TNF-α producing cells are found to be low in control (IgG) treated mice (~1 and 0.5%, respectively), as compared to αTGF-β treated (~2–3%) and αEBI-3 (~4–5%) treated group in mice (Figures 5A,B). Administration of αEBI-3 in combination with αTGF-β increased the IFN-γ and TNF-α producing cells significantly (~7.5 and 6%, respectively), compared to controls. There was no prominent difference in IL-4 producing CD4 cells post αCD25 or αTGF-β treatments (Figure 5C).

**Figure 6:**
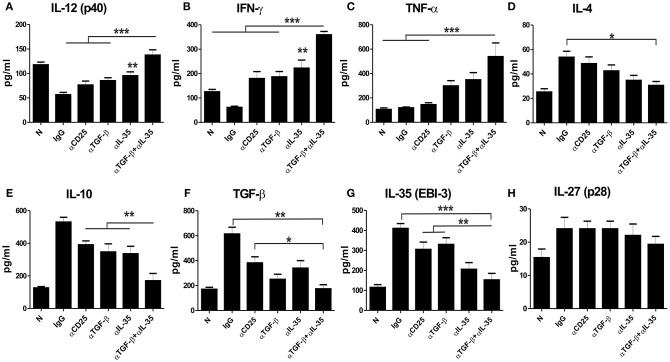
Cytokine responses post administration of neutralizing antibodies in the infected mice. LAg stimulated splenocytes of naive and infected mice were treated with neutralizing mAbs (as described in Materials and Methods) and cultured for 72 h. Culture supernatants were used for measuring IL-12 (p40) **(A)**, IFN-γ **(B)**, TNF-α **(C)**, IL-4 **(D)**, IL-10 **(E)**, TGF-β **(F)**, EBI-3 **(G)** and IL-27 (p28) **(H)** through Cytometric Beads Assay (CBA). Data expressed as means ± SE for five mice per group are representative of two independent experiments with similar results. *P < 0.05; **P < 0.01; ***P < 0.001 as assessed by one-way ANOVA and Tukey's multiple comparison tests.

We estimated various cytokines from the culture supernatants of splenocytes of αEBI-3, αTGF-β, and αEBI-3 mAbs treated mice through Cytometic Bead Assay (CBA). We found that infected mice treated with IgG produced low amounts of IL-12 (p40), IFN-γ, and TNF-α, while the mice treated with αEBI-3 or αTGF-β showed a significant increase in these cytokines. Administration of αEBI-3 alone or in combination with αTGF-β resulted in significant production of these pro-inflammatory cytokines (Figures 6A–C). Moreover, there was significant suppression of IL-4 and IL-10 responses along with TGF-beta and EBI-3 (Figures 6D–G). However, introduction of αEBI-3 along with αTGF-β not only resulted in the maximum production of these immuno-protective cytokines but also led to inhibition of disease promoting IL-4 and IL-10. No significant alteration in IL-27 (p28) levels in the culture supernatants of various indicated groups of mice (Figure 6G) were found. The effect of combined neutralization of TGF-β and EBI-3 on signaling cascade was also investigated. Previous reports showed that *Leishmania* infection is associated with enhanced expression of Arginase 1 and diminished production of nitric oxide synthase thereby low generation of the key anti-leishmanial molecule nitric oxide (25). The expression of arginase 1 from the splenic cell lysates derived from mice treated with the neutralizing antibodies for EBI-3, TGF-β, and CD25, was found to be down regulated as compared to control IgG-treated (Figure S1A). Mice treated with αCD25 only also showed a substantial reduction in arginase 1 expression compared to naive but the mice treated with αTGF-β+ α EBI-3 antibodies showed the best results with lowest expression of arginase 1. The antagonistic effect of arginase 1 to inducible nitric oxide synthase (iNOS) was found with significant loss of iNOS expression in the αTGF-β and αEBI-3 treated mice (Figure S1A). To investigate whether the reduction of arginase 1 and concurrent up regulation of iNOS is dependent on the STAT6 as reported by Osorio et al. during *L. donovani* infection (26) *in vitro* blocking of EBI-3 in the culture of splenocytes of infected mice was assayed. Neutralization of EBI-3 of infected splenocytes resulted in down regulation in the expression of both STAT6 as well as arginase 1 (Figure S1B).

### Neutralization of EBI-3 Along With TGF-β Limits Progression of *L. donovani* Infection in BALB/c Mice

**Figure 7:**
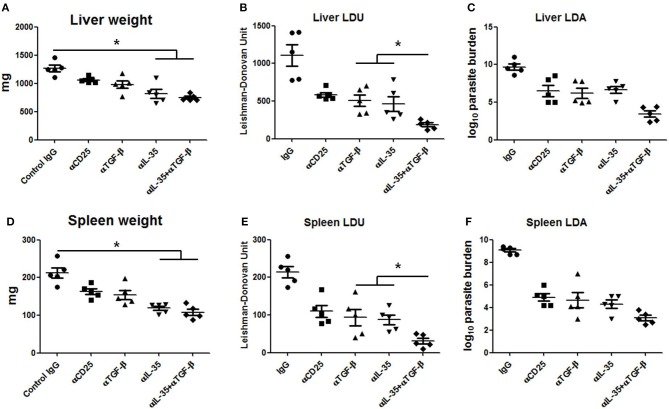
Parasite burden in neutralizing antibody treated infected mice. Infected BALB/c mice injected with 200 μg of neutralizing antibodies against TGF-β, IL-35, CD25, and isotype control (IgG cocktail) into the peritoneal cavity (as described in Materials and Methods) were sacrificed after 3 months of infection for measuring parasite burden in the liver and spleen. Total organ weights of liver and spleen **(A,D)** as well as their parasite burden were determined by LDU **(B,E)** and LDA **(C,F)**. Data expressed as means ± SE for five mice per group are representative of two independent experiments with similar results. *P < 0.05 as assessed by one-way ANOVA and Tukey's multiple comparison tests.

Since EBI-3 and TGF-β suppressed the host protective anti-leishmanial response, the effects of EBI-3 and TGF-β neutralization on disease progression in BALB/c mice were studied. A significant fall in the parasite burden of spleen and liver in the all treatment groups was observed when compared to control (Figures 7B,C,E,F). However, the most effective protection was observed in mice treated with a combination of αTGF-β + α EBI-3 mAbs. The reduction in parasite burden was concomitant to the resistance to hepato- and splenomegaly in the treatment groups (Figures 7A,D).

## Discussion

An impaired cellular immunity is a distinctive feature of active visceral leishmaniasis. Despite advances, the functional role of regulatory cells and their cytokines in suppression of anti-leishmanial immunity is not well elucidated. In this present study we have investigated the dynamicity of cellular and cytokine responses during experimental visceral leishmaniasis, and to determine the functional effects of the infection induced cytokines on progression of *L. donovani* infection in BALB/c mice.

Chronic infection of *L. donovani* in the mammalian phagocytes results in adenopathy of liver and spleen with loss of cell mediated immune responsiveness (19, 27, 28). A clear understanding of the immunology of VL primarily suffers due to limitations of appropriate disease models for immunological studies. Previous studies on infection kinetics of *L. donovani* AG83 in BALB/c mice indicate that similar to natural infection the disease is chronically progressive peaking up to 16 weeks post challenge (29, 30). Therefore, the same infection model was selected for the present study. The kinetics of parasite burden post challenge indicates a steep increase in liver infection at 2 months compared to splenic infection. Possibly, a complex cellular organization of spleen (12) contributes to initial resistance as observed during several disease models of acute VL infection (31). The progression of infection is suggestive of an acute pre-established infection at 1 month, established infection following 2 months and a chronic phase post 3 months of challenge with virulent *L. donovani* in BALB/c mice. Therefore, for studies related to comparisons of immunomodulations at pre-established (1 month) and extensively chronic (4 months) of infection time points were evaluated. However, *in vivo* neutralization studies were performed at 3 months of optimum chronic phase of infection. It is noteworthy that at early infection there was an enhanced induction of pro-inflammatory cytokine response of TNF-α and IFN-γ, which gradually reduced to basal levels with the surge of regulatory cytokines, IL-4, IL-10, and TGF-β in later infection. Studies on different subunit chains of IL-12 family revealed that p35 chain of IL-12 and EBI-3 were gradually up regulated, while p28 of IL-27 and p40 of IL-12 remained restricted during the course of infection. IL-12 (p35) and EBI-3 constitute IL-35, a cytokine known for its immunosuppressive role in cancer and other chronic infectious diseases, but is crucial for deterrence of autoimmune complexities (32–34). Previous reports implicate the involvement of IL-10 and TGF-β in restriction of the protective cytokine response in murine VL (27, 35, 36). The roles of IL-35 in VL pathology alone or in combination with other regulatory cytokines in immunomodulation have not been studied before. The surge in the levels of both the subunit chains of IL-35 in chronic *L. donovani* infection prompted an investigation on the cellular source and functional correlation of the cytokine. However, a major obstacle in the study of the fully functional IL-35 is the issue of cross-reactivity with either IL-12 or with IL-27. To address the issue the functional studies were limited to a single subunit chain, EBI-3 with parallel comparison with p28 (IL-27).

Immuno-phenotypic characterization of splenic T cells demonstrated a significant surge in proportions of CD4^+^CD25^+^ and CD4^+^FoxP3^+^ T accompanied with loss of cellular proliferative and proinflammatory cytokine response as chronic infection was established. Interestingly, at early infection a significant proportion of IFN-γ and TNF-α producing cells predominantly from CD4^+^CD25^−^ and CD4^+^FoxP3^−^ T cell were observed. However, with progression of infection the level of the proinflammatory cytokine producing cell populations were significantly reduced. The observations were in consistence to previous reports of anti-leishmanial response at acute phase of infection gradually progressing to T cell anergic state in chronic VL (37, 38). The drop in IFN-γ and TNF-α producing cells at chronic infection were accompanied with surge in the IL-10, TGF-β and EBI-3 producing CD4^+^ T cells. The functional association of cytokine response was analyzed by a combination of neutralization of IL-27 (p28), IL-10, TGF-β, and EBI-3 *in vitro* in the co-cultures of enriched CD4^+^CD25^+^ on CD4^+^CD25^−^ cells. Restoration of the effector functions and proliferation of co-cultured T cells following neutralization of IL-10, TGF-β and EBI-3 revealed the immuno-suppressive effect of the cytokines. IL-10 and TGF-β producing CD4^+^ T cell have been previously reported to dampen host inflammatory responses and to be involved in the pathogenesis of *L. donovani* infection (11, 12, 39, 40). In consistence with previous reports of induction of Th1 type immune response following IL-10 neutralization (41, 42), similar immunomodulatory effects were found in the *ex vivo* experiments. Our study extended the findings with EBI-3. Moreover, combined neutralization of TGF-β and EBI-3 resulted in the most effective restoration of proliferation and IFN-γ and TNF-α response. Unaltered immuno-repressed milieu post IL-27 (p28) blocking, either alone or in combination, indicated that IL-27 (p28) may not have a role in suppression of anti-leishmanial immunity. Therefore, it appears that EBI-3 dominantly from IL-35 plays a crucial role in immune suppression.

Earlier studies reported that IL-35, similar to IL-10, to be crucial for suppressing inflammatory responses during auto-immunity and cancer (13, 43, 44). Therapy with IL-35 during collagen-induced arthritis helped in the expansion of Tregs (45). During viral infection, targeting EBI-3 led to cure (14). The suppressive activities of IL-10 (46, 47) and TGF-β (48–50) and their contribution in murine VL are well established (51). Subsequently, Murray and colleagues proposed targeting IL-10 by neutralizing antibodies as a promising immunotherapy against murine VL (22, 46). To ascertain whether blocking the effect of TGF-β and EBI-3 *in vivo* can similarly resist *Leishmania* infection induced immune suppression, immunomodulatory effect and parasite burden of infected BALB/c mice treated with combinations of α-TGF-β and α-EBI-3 were evaluated. The *in vivo* neutralization led to significant increase in IL-12 (p40) and IFN-γ production post EBI-3 blocking along with a reversal in immune suppressed milieu by simultaneous neutralization of TGF-β and EBI-3 in infected mice. Blocking of these cytokines not only enhanced the pro-inflammatory cytokines but also increased the IFN-γ and TNF-α producing CD4^+^ T cells. Moreover, whereas, single neutralization of TGF-β or EBI-3 helped in partial resistance to the infection, their combined neutralization led to ~5-fold reduction in parasite load in both liver and spleen.

The resistance to parasite burden was also associated with a down regulation of arginase 1 expression, along with enhanced nitric oxide production. Suppression of arginase 1, an inhibitor of nitric oxide production (24) may result in NO mediated parasite killing. Moreover, this reduction of arginase 1 level was dependent on the expression of STAT6, which indicates that EBI-3 promotes *L. donovani* mediated arginase 1 expression in STAT6 dependent manner to down regulate NO production. Our studies, therefore, propose that EBI-3 is crucial for immunosuppression in VL. Moreover, EBI-3 together with TGF-β is the major contributor to the immunologically subverted milieu conducive for parasite survival and immune evasion.

## Conclusion

Progressive *L. donovani* infection in BALB/c mice results in gradual up regulation of IL-10, TGF-β and both the subunit chains of IL-35. The loss in proinflammatory response during chronic VL is accompanied by accumulation of CD4^+^CD25^+^ and CD4^+^FoxP3^+^ T cells with TGF-β and IL-35 (EBI-3) as the key factors for subversion of anti-leishmanial immunity. A more detailed study on the role of IL-35 and the subunit chains of IL-12 family may open new insights in understanding the host-pathogen immunology that can potentially generate novel immunotherapeutic approaches.

## Ethics Statement

The studies were performed according to the Committee for the Purpose of Control and Supervision on Experimental Animals (CPCSEA), Ministry of Environment and Forest, Govt. of India, and approved by the Animal Ethics Committee (147/1999/CPSCEA) of Indian Institute of Chemical Biology.

## Author Contributions

MA and NA: conceptualization. MA, NA, AS, and MS: methodology. MA, MS, AS, MK, SD, and ND: investigation. MA, AS, and NA: writing—original draft. MA, MS, AS, MK, and NA: writing—review and editing. NA: funding acquisition, resources, and supervision.

### Conflict of Interest Statement

The authors declare that the research was conducted in the absence of any commercial or financial relationships that could be construed as a potential conflict of interest.
