# Corrigendum: EBI-3 Chain of IL-35 Along With TGF-β Synergistically Regulate Anti-leishmanial Immunity

**DOI:** 10.3389/fimmu.2019.02409

**Published:** 2019-10-18

**Authors:** Mohammad Asad, Abdus Sabur, Mohammad Shadab, Sonali Das, Mohd. Kamran, Nicky Didwania, Nahid Ali

**Affiliations:** Infectious Diseases and Immunology Division, Council of Scientific and Industrial Research-Indian Institute of Chemical Biology (CSIR-IICB), Kolkata, India

**Keywords:** regulatory T cells, *Leishmania*, immune response, interleukin-35, transforming growth factor beta, immune suppression

In the original article, there was an error. “EBI-3” was misspelled as “EB1-3” in the title of the manuscript, supplementary material, and in the citation.

A correction has been made to the ***Title***:

“EBI-3 chain of IL-35 along with TGF-β synergistically regulate anti-leishmanial immunity”

Additionally, “EBI-3 and p35 chains of IL-35” was mistakenly referred to as “EBI-3 and p35 chains of EBI-3.”

A correction has been made to the ***Abstract***:

“Immunosuppression is a characteristic feature of chronic leishmaniasis. The dynamicity and the functional cross talks of host immune responses during *Leishmania* infection are still not clearly understood. Here we explored the functional aspects of accumulation of immune suppressive cellular and cytokine milieu during the progression of murine visceral leishmaniasis. In addition to IL-10 and TGF-β, investigation on the responses of different subunit chains of IL-12 family revealed a progressive elevation of EBI-3 and p35 chains of **IL-35** with *Leishmania donovani* infection in BALB/c mice. The expansion of CD25 and FoxP3 positive T cells is associated with loss of IFN-γ and TNF-α response in advanced disease. *Ex-vivo* and *in vivo* neutralization of TGF-β and EBI-3 suggests a synergism in suppression of host anti-leishmanial immunity. The down-regulation of EBI-3 and TGF-β is crucial for re-activation of JAK-STAT pathway for induction as well as restoration of protective immunity against *L. donovani* infection.”

The authors apologize for these errors and state that they do not change the scientific conclusions of the article in any way. The original article has been updated.

