# Supplementary material for: EBI-3 Chain of IL-35 Along With TGF-β Synergistically Regulate Anti-leishmanial Immunity
Source: Front Immunol. 2019 Apr 12;10:616. doi: 10.3389/fimmu.2019.00616 (PMC6474326; doi:10.3389/fimmu.2019.00616)
Supplement: Supplementary file 1 [file Data_Sheet_1.PDF]

Supplementary material

EBI-3 Chain of IL-35 Along With TGF-β Synergistically Regulate Anti-leishmanial Immunity

Mohammad Asad†, Abdus Sabur, Mohammad Shadab, Sonali Das, Mohd. Kamran,
Nicky Didwania and Nahid Ali*

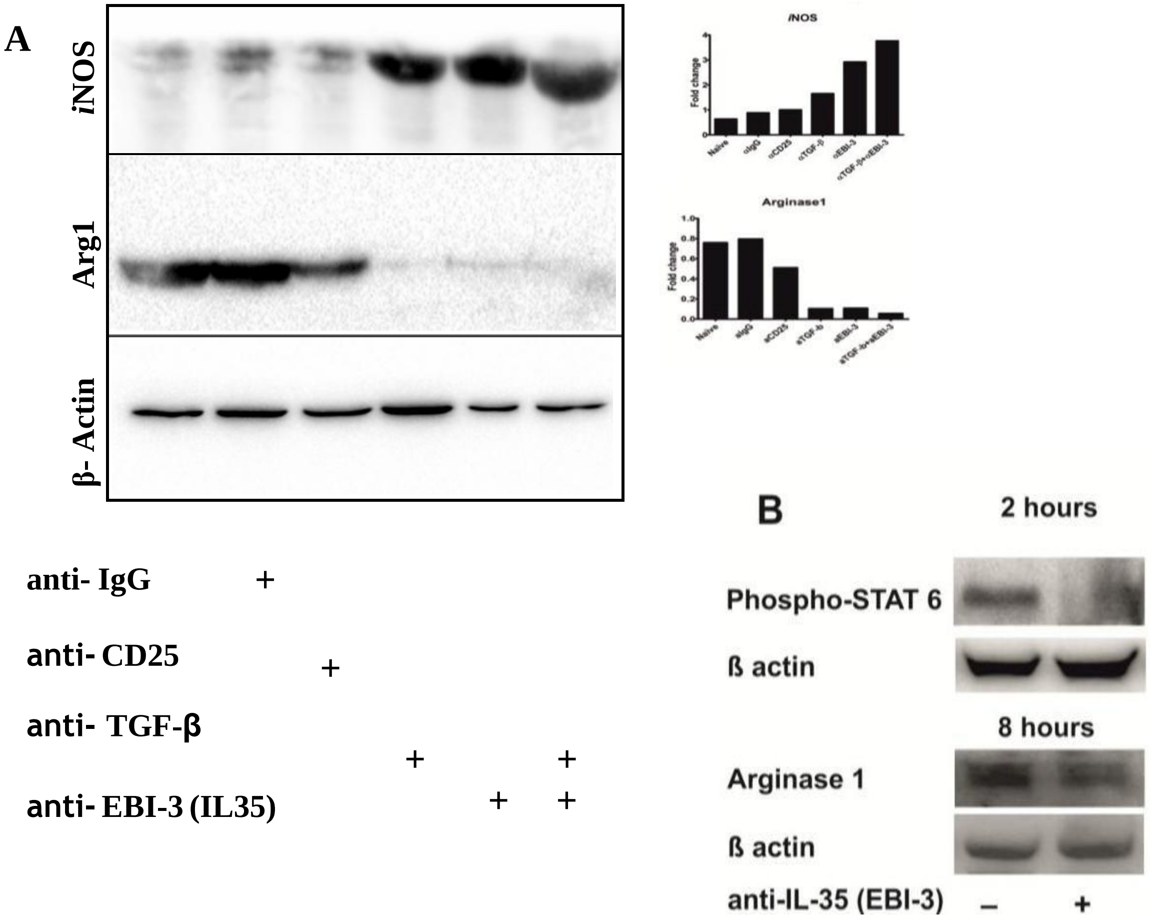

Figure S1. STAT6 dependent arginase 1 expression and NO production from the splenocytes of various neutralising antibodies treated mice. (A) Cell lysate was prepared from various antibody treated mice splenocytes' overnight culture and western blotting was done for arginase 1 and iNOS expression. (B) Culture of infected splenocytes were treated with αIL-35 (EBI-3) (20 μg/ml) and cells were collected for western blot at indicated time points. Level of expression of STAT6 and arginase 1 were measured through western blotting.
